# Differences in Cardiac Etiologies for Ischemic Stroke in Young and Middle-Aged Patients: A Single-Center Experience in Taiwan

**DOI:** 10.3390/jcm11082120

**Published:** 2022-04-11

**Authors:** Wen-Hwa Wang

**Affiliations:** 1 Department of Cardiology, Kaohsiung Veterans General Hospital, Kaohsiung 813, Taiwan; passtheboard1@gmail.com; 2 Health Management Center, Kaohsiung Veterans General Hospital, Kaohsiung 813, Taiwan; 3 Institute of Management, I-Shou University, Kaohsiung 840, Taiwan

**Keywords:** ischemic stroke, echocardiogram, cardioneurology

## Abstract

Background: The cardiac etiology of acute ischemic stroke (AIS) plays an important role in young adults; therefore, complete cardiac workups and subsequent prevention methods are important for treating young AIS patients. However, the definition of a young age for AIS patients could be below 45 years old, while in some previous studies, it has been below 55 years old. It remains unclear whether cardiac workups are equally important for AIS patients in the young (the age of 20–45 years old) and middle-aged (46–55 years old) categories. Materials and methods: This prospective study included 103 patients admitted due to a first AIS attack younger than 55 years old during the period from 1 October 2018 to 31 December 2020. All the patients received cardiologist consultations and cardiac workups accordingly. The characteristics of patients, cardiac workups, clinical findings, and management were analyzed. Results: AIS patients in the 46–55-year-old group had a higher prevalence of hypertension (61.4% vs. 39.0%), diabetes mellitus (27.3% vs. 15.3%), a previous history of coronary artery disease (9.1% vs. 1.7%), and atrial fibrillation (9.1% vs. 1.7%) compared to the 20–45-year-old group. After cardiologist consultations, a higher prevalence of newly diagnosed coronary artery disease (6.8% vs. 1.7%) and congestive heart failure (11.4% vs. 1.7%) was noted. Both groups disclosed similar percentages of patent foramen ovale (PFO) (27.3% vs. 22.0%) and valvular disease. These results led to subsequent changes in treatment in both groups. The 20–45-year-old group had higher percentages of receiving PFO occluders (11.9%) compared to the 46–55-year-old group (6.8%). Conclusion: Cardiologist consultations with thorough cardiac workups for AIS patients can reveal many cardiac findings in both young and middle-aged patients. This leads to a subsequent change in treatment, including medical and surgical aspects, which are important as secondary prevention for AIS.

## 1. Introduction

The incidence of acute ischemic stroke (AIS) has increased globally [1], as well as in young adults (<55 years old). [2,3,4] The recurrence of AIS is common [5,6,7] and leads to worse consequences for patients and their families and in the health care system. Identifying the correctable etiologies and reducing risk factors through thorough surveillances are mandatory for the secondary prevention of recurrent AIS. [6]

The etiologies and risk factors for AIS are heterogenous. Investigating the risk factors of AIS in young and old patients requires different methods. Cardiac origin emboli are one of the major sources of AIS in young adults. [8] Thus, extensive cardiologic evaluations, such as transesophageal echocardiography (TEE) or stress tests, are recommended for managing young stroke patients as needed. 

However, the definition of young stroke has not yet reached a consensus. Many studies have defined young stroke as the onset of an attack at under 45 years old, while some have considered the age of 55 as the cutoff point [9]. Since the AIS group between 46 and 55 years old is a gray zone, it is not conclusive whether comprehensive cardiac survey is beneficial to prevent AIS recurrence in this age group.

The aim of the study was to compare the characteristics of young stroke between different age groups, as well as the necessaries of cardiac workups for them. The secondary objective was to investigate the change in treatment after TEE.

## 2. Materials and Methods

### 2.1. Inclusion and Exclusion Criteria

This prospective study was approved by the Institutional Review Board of Kaohsiung Veterans General Hospital (VHGKS19-CT7-11). The inclusion criteria were adults (≥20 years old) who experienced a first AIS younger than the age of 55, who were admitted from 1 October 2018 to 31 December 2020 and were willing to visit the cardiology clinic after being discharged. 

The diagnosis of AIS was confirmed by brain computed tomography or brain magnetic resonance imaging. This study excluded those who refused to visit the cardiology clinic, after stroke caused by head trauma, and procedures, such as carotid endarterectomy, angiography procedure, or surgery. Patients with autoimmune diseases (such as systemic lupus erythematosus and Sjogren’s disease) and congenital coagulation disorders were also excluded.

### 2.2. Methods

The basic characteristics, comorbidities, and Modified Rankin Scale (mRS) were recorded. Patients had received a tentative survey, including carotid artery sonography, electrocardiogram (ECG), 24-h Holter monitor, and transthoracic echocardiography (TTE) routinely during the admission for AIS. Cardiac consultations were routinely arranged within 1–2 days post-stroke, and further cardiac workups, such as a stress test, thallium-201 scan, or coronary computed tomography angiography, were performed if coronary artery disease was suspected based on clinical judgement for individual cases. Patients visited the cardiac clinic and underwent TEE examination one week after being discharged. At the cardiology clinic, TEE with agitated saline (Philip iE 33 Cardiac Ultrasound machine or Philips EPIQ 7 Ultrasound) was performed for all patients, focusing on cardiac morphology, intracardiac shunt, or intracardiac emboli. Further cardiac interventions were performed according to the results of the cardiac workups afterwards. 

### 2.3. Statistical Analysis 

This study tabulated demographic, clinical variables, and cardiac interventions. Parameters are represented by percentages for categorical data. Parameters are signified by the mean and standard deviations for continuous data. A Chi-square test was used to compare categorical variables between groups. If the number in any cell was less than 5, Fisher’s exact test was used. An independent t-test was used to compare the difference between groups. Two-sided tests were used for analysis, and *p* < 0.05 was considered to be statistically significant. SPSS (IBM Corp. Released 2011. IBM SPSS Statistics for Window, Version 20.0. Armonk, NY, USA) was used for analysis. 

## 3. Results

### Patient Characteristics

There were 103 eligible patients with first-time AIS occurrence at the age of ≤55 years old. They were divided into two groups, namely, the 20–45-year-old group (*n* = 59) and the 46–55-year-old group (*n* = 44). There were no significant differences in the distribution of sex, severity of ischemic stroke (mRS scores), and BMI (Table 1).

Compared to the 20–45-year-old group, the 46–55-year-old group had a higher prevalence of hypertension (39.0% vs. 61.4%, *p* = 0.02) and current smoking (13.6% vs. 36.4%, *p* < 0.01), and a tendency of increasing prevalence of diabetes mellitus (15.3% vs. 27.3%), previous history of coronary artery disease (1.7% vs. 9.1%), and atrial fibrillation (1.7% vs. 9.1%), although there were no statistically significant results (Table 1).

After thorough cardiac workups arranged by cardiologists, further possible AIS-related diseases were diagnosed in both groups, such as coronary artery disease (1.7% vs. 6.8%) and congestive heart failure (1.7% vs. 11.4%). Meanwhile, an additional two cases (3.4%) had hypertrophic cardiomyopathy in the 20–45-year-old group.

Thirty-seven patients had abnormal findings in TEE, and twenty-seven of them (27/37, 73%) had unremarkable TTE findings. Most of those new findings were PFO, while others included AVM and bicuspid aortic valve. It is worth noting that TEE demonstrated further clear images and clinical implications, although those with abnormal findings had been revealed in TTE. Overall, TEE revealed that a high proportion of patients (32.2% vs. 36.4%) had obscure but important cardiac findings, which were potentially associated with AIS. The detailed numbers of each finding, including patent foramen ovale (PFO), aortic fibroelastoma, infective endocarditis, and myxoma, are listed in Table 2. Among them, PFO was the first common finding (22.0% and 27.3%). 

After cardiac workups, 16.9% and 15.9% of both groups received various cardiac interventions (Table 3), including PFO occluders (11.9% and 6.8%), coronary reperfusion therapy (1.7% and 4.5%), and valvular replacement (3.4% and 6.8%) (Table 3). During the follow-up time (up to 31 December 2021), seven patients (6.8%) were readmitted due to recurring stroke. Two of these patients, who were diagnosed with aortic papillary fibroelastoma and nonbacterial thrombotic endocarditis, refused surgical interventions initially but later experienced recurrent stroke. They both received surgical interventions afterwards.

## 4. Discussion

This study demonstrated that thorough cardiac examinations following cardiologic consultations disclosed a high prevalence of newly diagnosed cardiac diseases in young ischemic stroke patients, namely, the 20–45 and 46–55-year-old groups. Moreover, these findings led to the administration of secondary prevention by arranging cardiac interventions, such as PFO occluder implanting, valvular replacement, and coronary reperfusion therapy. This study found that routine cardiology consultation was necessary and beneficial for treating AIS in young adult patients.

On the other hand, when patients aged, especially for those older than 65 years old, the need for secondary prevention regarding underlying atherosclerosis, heart failure, metabolic syndrome, and arrythmia increased significantly. [10] Notably, this study revealed that conventional risk factors, such as hypertension, diabetes mellitus, and dyslipidemia, were not uncommon in the 46 to 55-year-old group. Unfortunately, most of the patients in this study were not aware of having these risk factors before the index admission of AIS. This implied that both patients and physicians did not realize that 46–55-year-old patients were vulnerable to conventional risk factors. Consequently, the population would not modify their lifestyles or take medication, and, eventually, this led to AIS. This study suggests that the age of 46–55 years old is an overlapping clinical zone mixed with other AIS risk factors for both younger (<45) and older patient groups (>55 years old). During the management of AIS in patients aged 46–55 years old, physicians should investigate conventional risk factors and arrange a more complete cardiovascular workup.

TEE provides a better opportunity to evaluate the morphology of the cardiac structure and detect the presence of intracardiac emboli. TEE can demonstrate the structure of PFO more sensitively and precisely in adult patients compared to TTE. However, TEE has not been recommended in routine practice, since the role of TEE has been viewed as controversial in AIS patients before. Although TEE played a significant diagnostic role, some studies have shown that there was not much of a change in the treatment plan, while others have shown that TEE could result in a treatment change. [11,12] In a recent multicenter study, Thomalla et al. included 454 patients diagnosed with AIS or transient ischemic attack with an undetermined cause from five different centers recruited over two years and concluded that TEE demonstrated a higher number of treatment-relevant findings than TTE for stroke patients [13]. Our results from a single-center study consisting of 103 AIS patients who received TEE were consistent with the study of Thomalla et al. Unlike their study, in which 191 (14.1%) patients were ≤60 years old with 27 (14.1%) treatment-relevant findings according to TEE, our study emphasized only AIS patients who were ≤55 years old. Among these patients, 27 (26.2%) treatment-relevant findings were discovered after performing TEE. Moreover, our study compared the importance of thorough cardiac workups between the 20–45- and 46–55-year-old groups.

On the other hand, there was a difference in smoking habits between the young and middle-aged AIS patients in this study. However, it did not largely affect the treatment-relevant findings of TEE, which were mainly congenital heart disease and valvular disease.

Our study proved that young and middle-aged AIS patients might have benefits after receiving TEE. Moreover, this study demonstrated a change in treatment afterwards: many patients received interventions, such as PFO occluders or valvular replacements. In addition, this study demonstrated the benefit of interdisciplinary teamwork with neurologists and cardiologists while treating AIS patients, especially for young adults. It is very important to involve cardiologists in evaluation in order to determine possible cardiac etiologies of those stroke patients. Sufficient evaluations led to correct diagnosis, adequate treatment and, subsequently, different long-term outcomes. For example, two studies argued that Julius Caesar’s epilepsy, with different cerebrovascular origins, severely affected his judgement and personality, which eventually led to his assassination. If he had been correctly evaluated by suitable specialists, he might have received adequate management. The history of the world would be different as he would have lived longer [14,15].

## 5. Limitations

First, generalizability might be a concern, since the study was based on a single center with a relatively small sample size. However, single-center studies have the advantage of reducing certain biases, since the neurologists and cardiologists have less heterogeneity in the practice protocol, as well as fewer regional differences in patient characteristics. In the future, the study design will be broadened and applied in other centers. Second, the included patients might not provide an overall representation of young AIS patients because those who refused to visit the cardiac clinic were excluded. This might have caused a selection bias. However, cardiovascular risks and structural heart diseases are not known factors affecting patients’ willingness for clinical referral. Third, the lack of data on the long-term outcomes of reducing recurrent stroke via TEE and cardiovascular consultation, due to the relatively small sample size, might be an issue. Nevertheless, several phase 3 studies demonstrated that treating underlying cardiac abnormalities, such as PFO, reduced recurrent stroke in ischemic stroke patients. It is important to perform a large-scale study to evaluate long-term outcomes.

## 6. Conclusions

An increasing number of studies, including the one described herein, have focused on the relationship between the brain and heart. It is very important to promote interdisciplinary teamwork between neurologists and cardiologists regarding the thorough cardiac evaluation and management of AIS patients, especially for young adults. Cardiologic consultation and TEE should be included in the regular workups. We should pay more attention to the 46 to 55-year-old group to investigate the etiology not only of conventional risk factors but also of structural heart disease, including PFO.

## Figures and Tables

**Table 1:** The demographic characteristics and associated factors of all subjects.

	20–45 y/o *n* = 59 (100%)	46–55 y/o *n* = 44 (100%)	*p*-Value
male sex	44 (74.6)	35 (79.5)	-
BMI	25.58 +/− 3.99	26.18 +/− 5.82	0.56
mRS score			0.59
0–1	33 (55.9)	21 (47.7)	-
2–3	19 (32.2)	15 (34.1)	-
4–5	7 (11.9)	8 (18.2)	-
**conventional CV risk factors**			
hypertension *	23 (39.0)	27 (61.4)	0.02
diabetes mellitus	9 (15.3)	12 (27.3)	0.13
CAD history	1 (1.7)	4 (9.1)	0.16
dyslipidemia	27 (45.8)	25 (56.8)	0.27
atrial fibrillation	1 (1.7)	4 (9.1)	0.16
PAOD	0 (0)	1 (2.3)	0.43
current smoking **	8 (13.6)	16 (36.4)	<0.01

* <0.05, ** <0.01; CAD: coronary artery disease; mRS: modified Rankin Scale; PAOD: peripheral artery occlusion disease; y/o: years old.

**Table 2:** Findings of cardiovascular consultation and TEE.

	20–45 y/o	46–55 y/o	*p*-Value
**Cardiovascular consultation**	4 (6.8)	8 (18.2)	0.74
CAD ^¶^	1 (1.7)	3 (6.8)	
CHF ^†^	1 (1.7)	5 (11.4)	
HCM	2 (3.4)	0 (0)	
**TEE findings**	19 (32.2)	18 (36.4)	0.36
PFO	13 (22.0)	12 (27.3)	
non-rheumatic valvular heart disease	0 (0)	1 (2.3)	
infective endocarditis	1 (1.7)	1 (2.3)	
nonbacterial thrombotic endocarditis	1 (1.7)	0 (0)	
bicuspid aortic valve	1 (1.7)	1 (2.3)	
prosthetic valve	0 (0)	1 (2.3)	
gerbode ventriculo-atrial defect	1 (1.7)	0 (0)	
AVM	1 (1.7)	0 (0)	
Myxoma	0 (0)	1 (2.3)	
aortic papillary fibroelastoma	1 (1.7)	1 (2.3)	

^¶^ CAD was newly diagnosed by cardiologic consultation. ^†^ Left ventricular ejection fraction (LVEF) < 35%. AVM: arteriovenous; CAD: coronary artery disease; CHF: congestive heart failure; HCM: hypertrophic cardiomyopathy; PFO: patent foreman ovale; TEE: transesophageal echocardiogram.

**Table 3:** Cardiac interventions after cardiologist consultations and TEE.

	20–45 y/o	46–55 y/o	*p*-Value
**Intervention for TEE findings**	10 (16.9)	7 (15.9)	0.89
PFO occluder	7 (11.9)	3 (6.8)	
valvular replacement ^†^	2 (3.4)	3 (6.8)	
myxoma excision	0	1 (2.3)	
gerbode defect occluder	1 (1.7)	0	
**Reperfusion therapy for CAD ^‡^**	1 (1.7)	2 (4.5)	

^†^ included aortic or mitral valve replacement. ^‡^ included percutaneous coronary intervention and coronary artery bypass graft.
